# Lipophilic statins inhibit Zika virus production in Vero cells

**DOI:** 10.1038/s41598-019-47956-1

**Published:** 2019-08-07

**Authors:** Erica Españo, Jeong-Hyun Nam, Eun-Jung Song, Daesub Song, Chong-Kil Lee, Jeong-Ki Kim

**Affiliations:** 1 Department of Pharmacy, Korea University College of Pharmacy, Sejong, 30019 Republic of Korea; 2 Department of Pharmacy, College of Pharmacy, Chungbuk National University, Cheongju, Chungbuk 28160 Republic of Korea

**Keywords:** Viral infection, Antivirals, Phenotypic screening

## Abstract

Zika virus (ZIKV) is a mosquito-borne member of the *Flaviviridae* family. ZIKV infection has been associated with neurological complications such as microcephaly in newborns and Guillain-Barré syndrome in adults; thus, therapeutic agents are urgently needed. Statins are clinically approved for lowering cholesterol levels to prevent cardiovascular disease but have shown potential as antiviral drugs. In this study, we explored the possibility of utilizing statins as anti-ZIKV drugs. We found that, generally, lipophilic statins (atorvastatin, cerivastatin, fluvastatin, lovastatin, mevastatin, and simvastatin) could reduce ZIKV production *in vitro* and result in smaller foci of infection. Time-of-drug-addition assay revealed that early treatment with statins is more beneficial than late treatment; however, statins could not completely inhibit the entry stage of ZIKV infection. Furthermore, individual lipophilic statins differed in anti-ZIKV capacity, with fluvastatin being the most efficient at low concentrations. Taken together, this study shows that statins or their derivatives have the potential to be used as anti-ZIKV therapeutic agents.

## Introduction

Zika virus (ZIKV) is an enveloped, positive-sense, single-stranded RNA virus belonging to the genus *Flavivirus* of the *Flaviviridae* family. The viral genome encodes a polyprotein consisting of the capsid (C), premembrane/membrane (prM), envelope (E), and seven nonstructural proteins (NS1, NS2A, NS2B, NS3, NS4A, NS4 B, and NS5)^1^. Like some of the clinically relevant members of the *Flaviviridae* family, such as Dengue virus (DENV), West Nile virus (WNV), Yellow fever virus (YFV), and Japanese encephalitis virus (JEV), ZIKV is arthropod-borne (i.e. an arbovirus); its main vectors for transmission are *Aedes* mosquitoes, primarily *A*. *aegypti*. Typically, ZIKV causes benign, febrile disease in around 18% of infected people^2^, with symptoms resembling those of other arboviral infections like dengue and chikungunya, namely fever, rash, arthralgia, conjunctivitis, and, less frequently, headache, vomiting, and jaundice^3^. For around 50 years since its discovery in 1947^4^, ZIKV infections in humans have been sporadic and have been limited to African and Asian countries where ZIKV is believed to be endemic. Large outbreaks, however, began to erupt outside Africa and Asia in 2004, starting from the Yap Island and extending to other Pacific Islands in succeeding years^2,5^. The largest known ZIKV outbreak to date started in 2014 in Brazil. It is believed to have affected 0.5–1.5 million people and triggered the spread to other South American countries and US territories^6^. Until this outbreak in Brazil, ZIKV has generally been considered to cause mild disease. With the high incidence rate, however, emerged convincing correlations of ZIKV infection with neurological disorders, namely microcephaly in newborns^7,8^, and Guillain-Barré syndrome (GBS) in adults^9,10^. The dangers associated with ZIKV led the World Health Organization (WHO) to declare a Public Health Emergency of International Concern (PHEIC) in February 2016^11^.

Although the ZIKV outbreak in the Americas has ended, the complications associated with ZIKV infection remain causes for concern, especially in light of possible ZIKV emergence in immunologically naïve populations. Therefore, countermeasures against ZIKV infection have to be established to prevent ZIKV pandemics and the complications that arise from infection. To date, there is no specific therapeutic agent, as is the case for most other flaviviruses, and no vaccine against ZIKV. Therefore, it is imperative to identify drugs that have the ability to combat ZIKV.

Statins are reversible inhibitors of 3-hydroxy-3-methylglutaryl-CoA (HMG-CoA) reductase (HMGCR), an enzyme involved in cholesterol biosynthesis^12^. They are clinically approved and used for reducing cholesterol levels to prevent primary and secondary cardiovascular disease. In addition, statins show a vast number of pleiotropic (i.e. cholesterol-independent) effects that contribute to their efficacy as therapeutic agents. Due to their wide range of action, statins have great potential to be repurposed for treatment of other diseases including cancer^13^, Alzheimer’s disease^14,15^, and multiple sclerosis^16^. Statins have also been reported to have antiviral activity against HCV^17^, DENV^18^, and influenza virus^19,20^. A primary screen for potential repurposing of FDA-approved drugs in multiple cell culture models showed that lovastatin has anti-ZIKV activity^21^, and in our own screening studies of a chemical library, mevastatin and simvastatin exhibited antiviral activity against ZIKV in Vero cells. These suggest that this class of drugs may have anti-ZIKV activity. Although statins share a pharmacophore, they vary greatly in their substituents, which may translate to differences in pharmacological activity. As such, we decided to explore the anti-ZIKV effects of different statins using Vero cells. We initially examined eight statins (lipophilic and hydrophilic) for possible anti-ZIKV activity in Vero cells. We found that lipophilic statins were able to reduce the production of infectious ZIKV particles in Vero cells, but there was variability in the anti-ZIKV activity of individual statins. Our results suggest that statins or their derivatives can potentially be developed as therapeutic agents against ZIKV.

## Results

### Lipophilic statins reduce ZIKV infectivity in Vero cells

**Figure 1:**
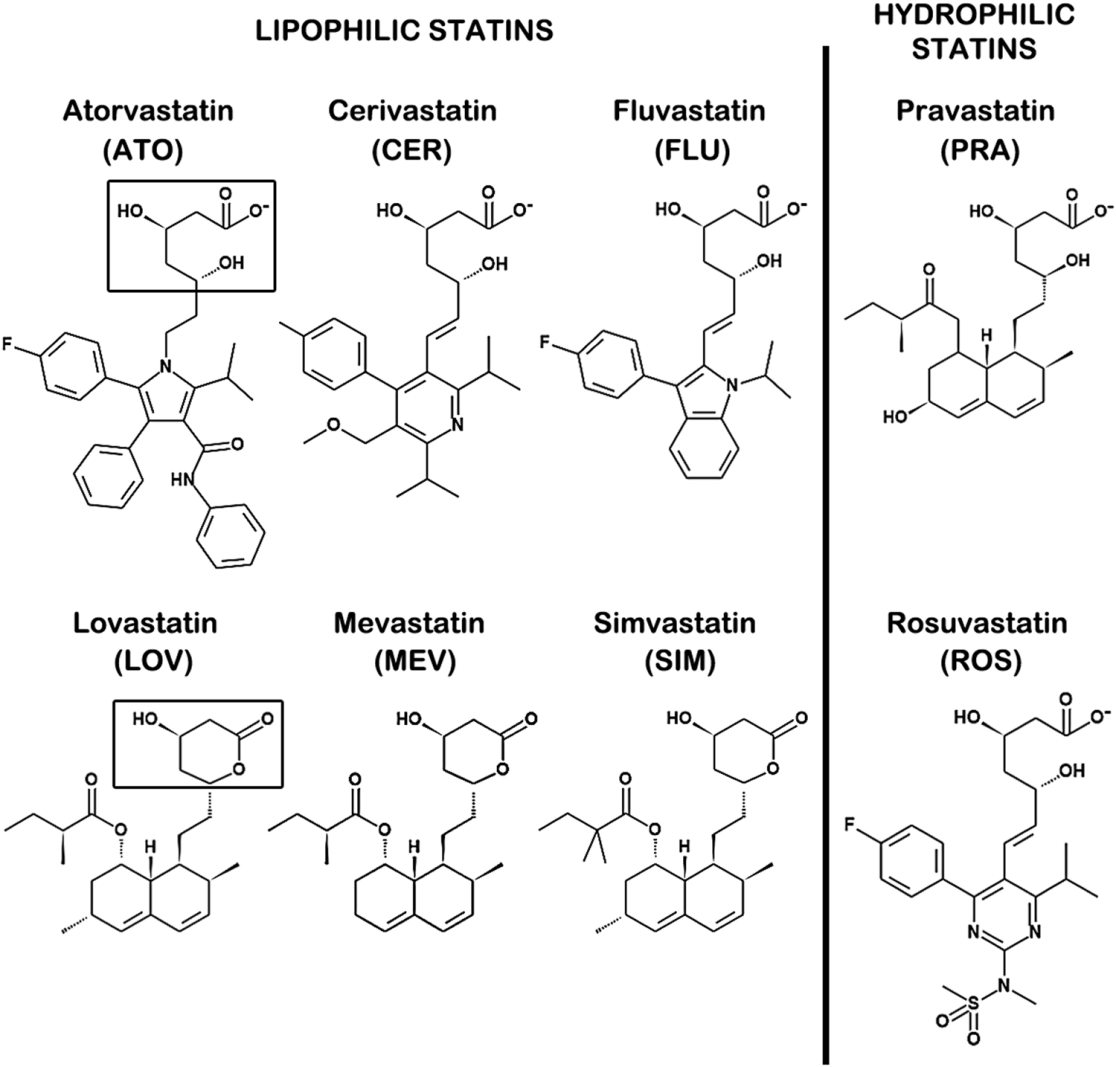
Statin chemical structures. The statins used in this study can be grouped into lipophilic and hydrophilic statins. Each statin has a pharmacophore (enclosed in boxes), that is either an open-ring (e.g. ATO) or a closed-ring (e.g. LOV), attached to a substituent ring system.

Eight statins, including atorvastatin (ATO), cerivastatin (CER), fluvastatin (FLU), lovastatin (LOV), mevastatin (MEV), pravastatin (PRA), rosuvastatin (ROS) and simvastatin (SIM) (Fig. 1), were initially examined for activity against ZIKV infection at a multiplicity of infection (MOI) of 0.01 and for cytotoxicity in Vero cells by a colorimetric cytotoxicity assay. Lipophilic statins (ATO, CER, FLU, MEV, LOV and SIM) inhibited ZIKV *in vitro*; their half maximal effective concentration (EC_50_) and cytotoxic concentrations (CC_50_) are shown in Table 1. Of these, CER had the lowest EC_50_ value while LOV had the highest, showing differences in pharmacological activity among the different statins. However, at the ranges tested, little to no inhibition was observed for the hydrophilic statins PRA and ROS at up to 50 and 25 µM, respectively, and apparent activity was observed at concentrations that were cytotoxic to Vero cells (see Supplementary Fig. S1); therefore, their EC_50_ and CC_50_ values could not be calculated.

**Table 1:** Anti-ZIKV activity (EC_50_) and cytotoxicity (CC_50_) of statins in Vero cells. SI, selectivity index, CC50/EC50; NC, not calculated.

Statin	EC_50_ (µM)	CC_50_ (µM)	SI
Atorvastatin (ATO)	7.34	24.31	3.31
Cerivastatin (CER)	0.02	0.37	18.19
Fluvastatin (FLU)	1.49	6.03	4.06
Lovastatin (LOV)	14.59	38.76	2.66
Mevastatin (MEV)	3.39	14.45	4.27
Simvastatin (SIM)	2.55	7.14	2.81
Pravastatin (PRA)	NC	NC	NC
Rosuvastatin (ROS)	NC	NC	NC

Our results suggest that the lipophilic statins used in this study have the capacity to inhibit ZIKV *in vitro*. However, our results do not necessarily mean that the hydrophilic statins are not capable of inhibiting ZIKV infection. Lipophilic statins readily enter cells through passive transport, while hydrophilic statins rely on active transport to enter cells. The hydrophilic statins may not be able to enter Vero cells, thus appearing ineffective; other cell culture models, especially those of hepatic origin (e.g. Huh-7), may instead be used to test the ability of hydrophilic statins to reduce ZIKV infection.

### Co-introduction of lipophilic statins with ZIKV reduces production of infectious virus particles in Vero cells

**Figure 2:**
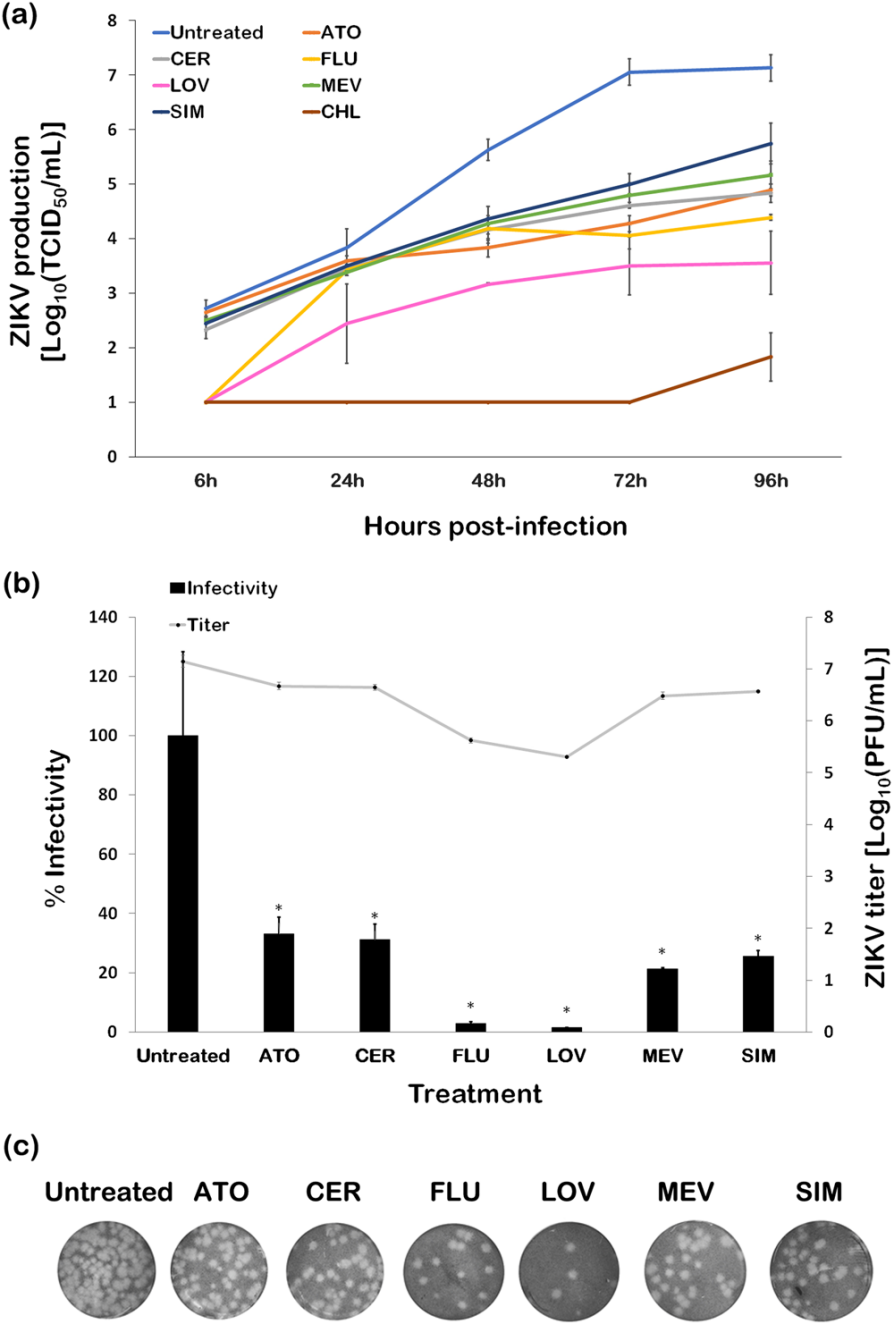
Inhibition of infectious ZIKV production by lipophilic statins in Vero cells. (**a**) The lipophilic statins and chloroquine (reference drug) were used to simultaneously treat Vero cells infected with ZIKV (MOI of 0.01). Culture supernatants were collected at different timepoints, and viral production (TCID_50_) was measured by observation of cytopathic effects (CPE) after 96 hours. (**b**) Culture supernatants were also subjected to plaque assay in Vero cells. Percent infectivity was calculated by dividing the titer of the statin-treated setup by that of the untreated titer (PFU/mL). (**c**) Comparison of plaques formed in Vero cells at 10^−5^ dilution of supernatants are shown. *p < 0.01, Student’s *t*-test), and data presented are means ± SEM.

We next observed the effects of statin treatment on the production of infectious ZIKV particles *in vitro* through time-based studies. We simultaneously added ZIKV (MOI of 0.01) and inhibitory but minimally cytotoxic concentrations of the lipophilic statins (14.69 µM for ATO, 91 µM for CER, 2.97 µM for FLU, 29.18 µM for LOV, 6.78 µM for MEV, and 5.09 µM for SIM; see Supplementary Fig. S2 for effects on cell morphology) to Vero cell cultures. Neither ZIKV nor statins were removed throughout the incubation period to account for possible effects of the drugs on the different stages of the ZIKV replication cycle. Culture supernatants were collected at different timepoints, and the ZIKV titers, represented as the median tissue culture infective dose (TCID_50_) based on cytopathic effects (Fig. 2a), were determined. All the statins were able to significantly reduce ZIKV production after more than 24 hours post-infection (hpi), and the decline was sustained till the end of infection (96 hpi). At the concentrations tested, FLU and LOV were able to reduce ZIKV production more effectively than the rest. Chloroquine (CHL; 25 µM) has been previously reported as a potential anti-ZIKV agent and was therefore used as a reference drug for activity against ZIKV^22,23^.

In order to support our observations in the timepoint assay, we co-introduced the lipophilic statins with ZIKV (MOI of 0.01) in Vero cells and determined the endpoint infectious ZIKV titers in the culture supernatant through the plaque assay (Fig. 2b,c). Production of infectious ZIKV particles in the statin-treated setups were significantly lower than in the untreated control, with infectivity reduced by a range of ~67 to 98%. Consistent with the observations made in the timepoint assay, FLU and LOV had the highest inhibition rates at the tested concentrations, reducing the infectious titer of ZIKV to 2.94% and 1.42% of the untreated control, respectively. These results show that statins are able to reduce the production of infectious ZIKV particles in Vero cells and that individual statins have different capacities for ZIKV inhibition.

### Statin treatment reduces intracellular ZIKV production in vero cells

**Figure 3:**
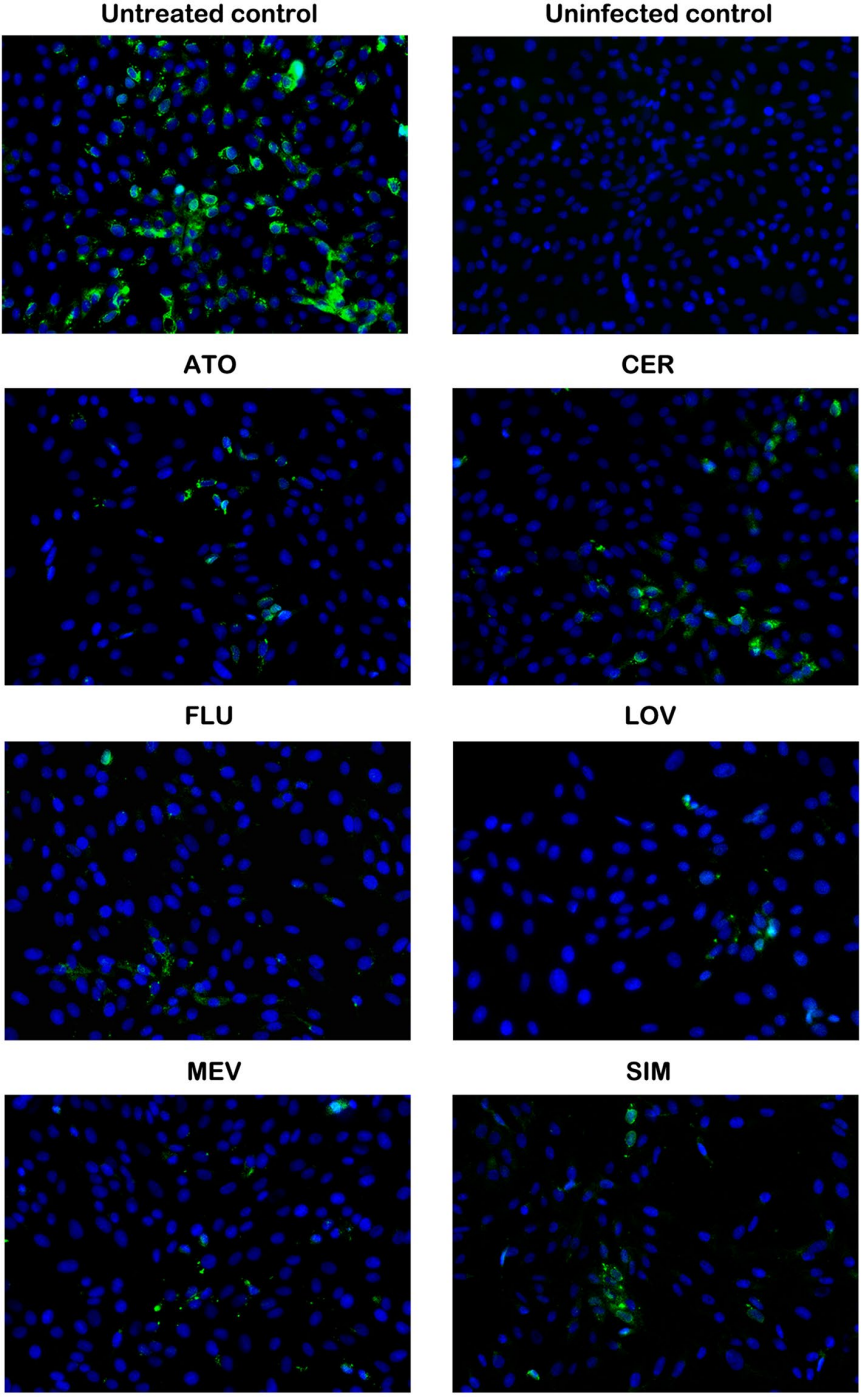
Lipophilic statins limit ZIKV localization in Vero cells. Cells were stained for flaviviral E-protein (4G2) using the 4G2mAb and counterstained with DAPI as a nuclear stain at 48 hpi (MOI of 0.1). ZIKV particles were spread in the perinuclear region and the cytoplasm of untreated cells; however, with statin treatment, ZIKV particles were generally confined to the perinuclear region.

To determine the effects of lipophilic statins on ZIKV within the cells, we adopted the same scheme for treatment on ZIKV-infected (MOI of 0.1) Vero cells and performed the immunofluorescence assay (IFA) against flaviviral E-protein (4G2) 48 hpi (Fig. 3). All the statin-treated cultures showed smaller foci of infection and lower proportions of infected cells than the untreated ZIKV-infected culture. The replication process of flaviviruses, which is also believed to apply to ZIKV, takes place in invaginations on the endomembrane system, particularly on the surface of the endoplasmic reticulum. In our study, replication could be observed in the perinuclear region of the cells. The untreated culture had numerous ZIKV particles within the cells (in the perinuclear region and all over the cytoplasm), suggesting that there was replication and trafficking of the virus. However, in the statin-treated infected Vero cells, ZIKV particles had a more limited subcellular localization. The ZIKV particles appeared to be confined to the perinuclear region in the cells in the statin-treated setups, with some occurring as perinuclear dots. These results imply that replication or trafficking was arrested in the infected cells. There were also visual differences in the effects of the various statins on the spread of the virus, wherein CER appeared to have the least inhibitory action on replication, as indicated by its larger foci of infection and wider localization of the virus. These results indicate that the statins may be able to reduce ZIKV infection either by inhibiting entry, as seen by the lower number of infected cells, or by inhibiting replication, as noted by the infected cells with limited ZIKV localization.

### Early addition of statins marginally reduced ZIKV production compared to late addition

**Figure 4:**
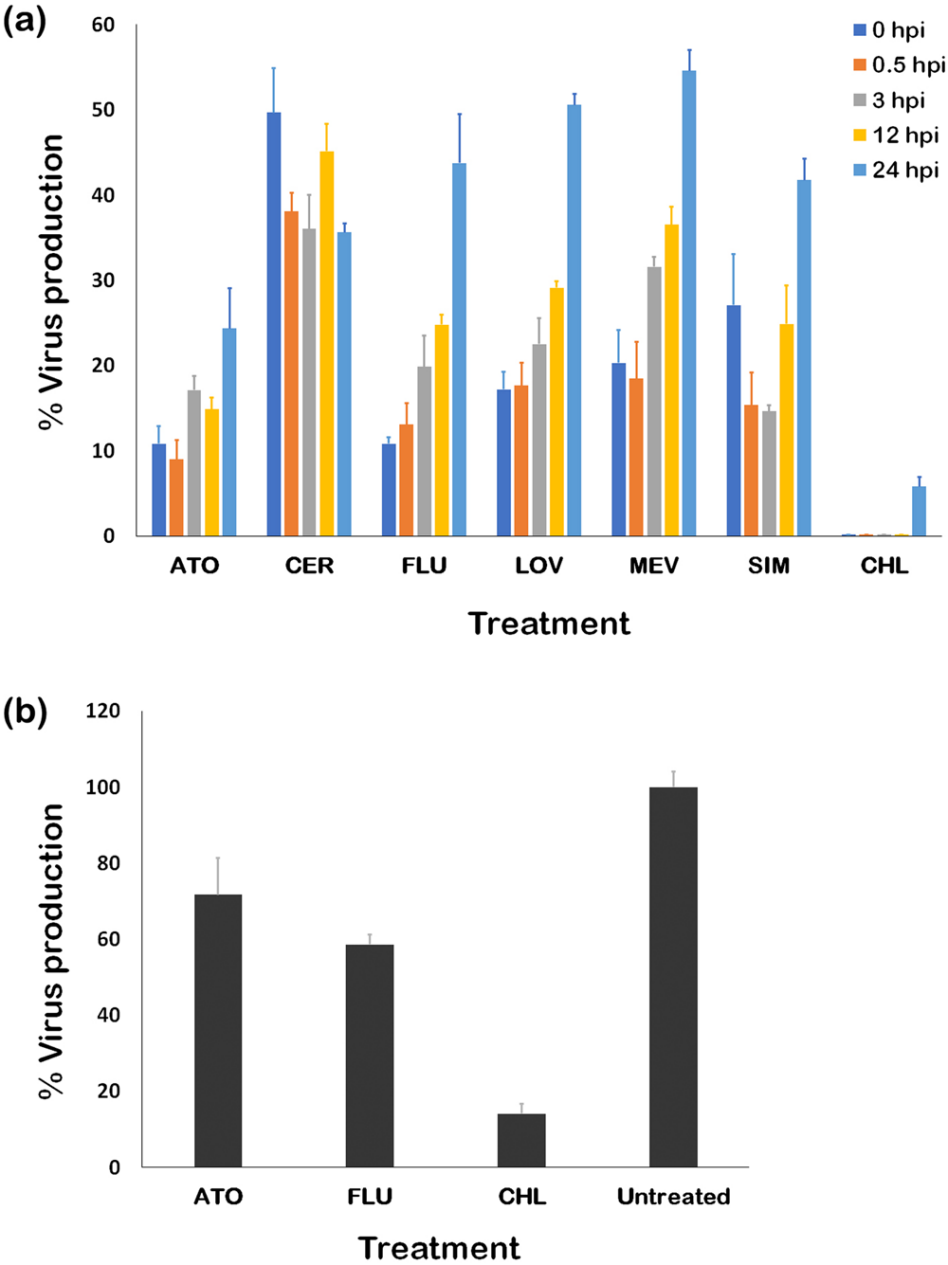
Early treatment with lipophilic statins is more effective than late treatment in reducing ZIKV in Vero cells. (**a**) ZIKV-infected (MOI of 5.0) Vero cells were treated with statins and chloroquine, a previously reported inhibitor of ZIKV entry, at different timepoints post-infection (0, 0.5, 3, 12 and 24 hpi). The plaque assay was performed on culture supernatants after 36 hours to determine endpoint ZIKV titers for each treatment. (**b**) Based on (a), representative statins (FLU and ATO) were chosen for the virus internalization assay to determine their effects on viral entry by treating ZIKV-infected (300 PFU) Vero cells with the drugs (1 hour), followed by the plaque assay. Data presented are means ± SEM.

To determine the possible target of statins in the ZIKV replication cycle, we performed a time-of-drug-addition assay. First, Vero cells were infected with ZIKV (MOI of 5.0) for 1 hour. The cells were washed, and media was replenished. Then the statins were added at different timepoints (0, 0.5, 3, 12, or 24 hpi; n = 3 per timepoint). Finally, the ZIKV the supernatants were collected at 36 hpi, and the endpoint titers were determined by the plaque assay (Fig. 4a). Significant reduction in ZIKV production was found for all statins at all timepoints (*p* < 0.001). We found that FLU, LOV and MEV had similar trends in inhibiting ZIKV production. Specifically, their effects peaked upon addition at 0 hpi and declined steadily over later timepoints of addition. This suggests that FLU, LOV and MEV target early stages of ZIKV infection. On the other hand, the effect of ATO peaked upon addition at 0.5 hpi and declined steadily when added at later timepoints. This suggests that ATO also targets an early stage in ZIKV biogenesis but with slight delay compared with the effects of FLU, LOV and MEV. Peak activity of SIM was observed upon addition at 3 hpi, which suggests that it targets an early-to-middle stage in ZIKV biogenesis. While reduction in ZIKV titers was observed for CER, there was no apparent trend in its effects; moreover, its effects were significantly less than the effects of the other statins (*p* < 0.001). The reference compound, CHL, significantly reduced ZIKV titers at all titers, but also had greater effects upon addition at 0–12 hpi than at 24 hpi. Taken together, our results suggest that statins affect early stages of ZIKV infection but with some differences between individual statins.

### Statins reduce ZIKV capacity for entry

Since the early addition of ATO and FLU had the greatest effects on ZIKV production, we determined the effects of these statins on ZIKV internalization. Vero cells in 6-well plates (~95% confluence) were infected with 300 PFU of ZIKV, allowed to adsorb at 4 °C for 1 hour, and treated with ATO and FLU for 1 hour after infection. Results of the viral internalization assay (Fig. 4b) show that both ATO and FLU were able to reduce plaque formation down to 71% and 58%, respectively, while CHL, which has been previously reported to inhibit ZIKV entry, reduced plaque formation to 14% compared to the ZIKV-infected control^23^. These results suggest that ATO and FLU were able to partially inhibit ZIKV entry.

## Discussion

Statins are reversible, selective inhibitors of HMGCR. As HMG-CoA analogs, they compete with HMG-CoA for the binding site of the enzyme, thereby disrupting the conversion of HMG-CoA to L-mevalonate^12^. This conversion is a rate-limiting step in the mevalonic acid (MVD) pathway. Therefore, statins impede downstream processes in the pathway, including the synthesis of cholesterol and the production of isoprenoid metabolites (e.g. geranylgeranylpyrophosphate and farnesylpyrophosphate). The statin pharmacophore is a modified hydroxyglutaric acid segment, either in a closed or open ring form, which is attached to a substituent ring system (Fig. 1). Variations in these substituents lend differences to the pharmacological properties (e.g. solubility, bioavailability and stability) of the statins. In this study, we initially investigated eight statins, six of which are more lipophilic (ATO, CER, FLU, LOV, MEV, and SIM) than the other two (PRA and ROS), for their activity against ZIKV *in vitro*.

The results of our study show that the use of statins lowers the production of infectious ZIKV particles in Vero cells. Specifically, treatment of ZIKV-infected Vero cells with lipophilic statins (ATO, CER, FLU, MEV, LOV and SIM) for the entire duration of infection results in decreased production of infectious ZIKV particles into the culture supernatant over time. This reduction can be as high as 98% as seen with LOV treatment. Furthermore, immunofluorescence assay revealed that statin treatment reduced the capacity of ZIKV to infect cells, resulting in lower proportions of infected cells and smaller foci of infection. The ZIKV particles also appeared to have more limited localization and were more confined to the perinuclear region in statin-treated cells compared to untreated, ZIKV-infected Vero cells. These results suggest that cholesterol or other products of the MVD pathway are important in the ZIKV replication cycle.

Although the ZIKV replication cycle has not yet been fully explored, it is believed to follow the typical flaviviral replication cycle. Flaviviral biogenesis begins when the E protein on the viral envelope binds receptors on the host’s plasma membrane. This is followed by clathrin-mediated entry; fusion of the virus and host cell membranes; and nucleocapsid release and disassembly. Replication of the viral genome takes place in virus-induced intracellular membrane packets, typically on the surface of the ER. Then, the immature flaviviral particles are packaged and assembled in invaginations on the endomembrane. The immature virions are subsequently shuttled out of the cells via the secretory pathway.

Several studies have been performed to identify the stages in flaviviral replication cycle where cholesterol is involved. In WNV, for example, sequestration of cholesterol from the plasma membrane resulted in lower viral titers and failed virus internalization, and that increased cholesterol levels promoted fusion of a liposome model with a target membrane^24–26^. These observations suggest that in some flaviviruses, cholesterol influences early stages of infection. The contribution of cholesterol at the entry stage is proposed to be due to the requirement of the viruses to attach to receptors prior to infection^24,27,28^. Receptors are concentrated within lipid rafts, which are cholesterol-rich assemblies on the plasma membrane^28^. Depletion of cholesterol will affect the assembly of receptors, thereby reducing chances of viral adhesion to the host. Cholesterol is also believed to play a part in the flaviviral replication stage. Infection with WNV has been reported to cause redistribution of cholesterol from the plasma membrane to the sites of replication^29^. Lipid rafts are hypothesized to be involved at this stage in order to increase the surface area available for viral replication and to concentrate the replication factors within the vesicle packets^28^. Indeed, DENV and JEV replication has been shown to occur in cholesterol microdomains or lipid rafts inside the cell^30^. The role of cholesterol in the early stages of flaviviral biogenesis may not be limited to binding, however, as the use of a cholesterol transport inhibitor did not prevent binding but disrupted trafficking of DENV inside infected cells^31^. Interestingly, another study in DENV demonstrated that disrupting cholesterol biosynthesis did not inhibit replication but resulted in lower virus production, indicating a role for cholesterol in later stages of viral biogenesis^32^.

As such, cholesterol may also be involved in several stages of the ZIKV life cycle, and one of these stages is likely to be the target of statins in ZIKV. Based on the results of our time-of-drug-addition assay, early addition (0–3 hpi) of statins to ZIKV-infected Vero cells causes significant decrease in virus production. However, the results of the internalization assay showed that the effects of 1-hour statin treatment (FLU and ATO) on ZIKV entry were not as profound as the effects of co-treatment with statins throughout the entire incubation period. On the other hand, treatment with chloroquine, an agent that has been reported to affect ZIKV entry, had a more marked effect on ZIKV internalization. Therefore, our results suggest that while statins can inhibit early stages of ZIKV biogenesis, statins cannot inhibit ZIKV entry completely. We hypothesize that, given the multiple stages where cholesterol is involved, statins affect multiple events in the ZIKV replication cycle. These effects then add up to a more dramatic reduction in ZIKV production compared to targeting only a single stage of ZIKV biogenesis. However, since we did not quantify intracellular viral load, we cannot state which other stages are affected by statins.

The anti-ZIKV activity of statins in this study is consistent with reports of statin activity against other flaviviruses. Statins have demonstrated the ability to inhibit infection of DENV^18,33^, and WNV^29^ *in vitro*, and statin treatment has attenuated DENV2 infection in mouse models^34^. Thus far, the most advanced use of statins as antivirals is against Hepatitis C virus (HCV), another member of the *Flaviviridae* family, where it is currently utilized as adjuvant to improve the standard of care treatment^35,36^. In these viruses, the effect of statins is mostly attributed to its ability to inhibit cellular synthesis of cholesterol, thereby highlighting the importance of cholesterol in flaviviral biogenesis. However, one study in DENV2, where statins were shown to inhibit viral assembly, suggested that the statins’ antiviral effect was not completely dependent on its anti-cholesterol activity^18^. Another study has likewise reported that the anti-DENV2 activity of statins was not due to its effect on cholesterol levels^37^. Given these conflicting reports on the specifics of statins’ antiviral activity, no specific target for statins in flaviviruses has been identified.

Additionally, statins have been reported to exhibit antiviral activity against viruses belonging to other families. Interestingly, statins have been found to inhibit entry and production of the human immunodeficiency virus (HIV) in a cell culture model by preventing geranylgeranylation of the Rho protein^38^. Statins have also been reported to inhibit Ebola virus (EBOV) infectivity by preventing the maturation of the EBOV glycoprotein^39^. Although the specific mechanisms by which statins affect EBOV glycoprotein maturation has not been identified, this study showed that the mechanism is not based on the statins’ anti-cholesterol activity and may instead be due to the statins’ effects on other products of the MVD pathway. These studies imply that the antiviral activity of statins is specific to a virus’ requirements for biogenesis. Furthermore, due to the multiple products of the MVD pathway and the consequent pathways affected by these products, statins may be able to target multiple stages in viral biogenesis, depending on the virus. Whether cholesterol or other products of the MVD pathway are affected by statins in ZIKV will have to be determined in future studies.

Similar to results of other studies, our results show that different statins have varying levels of antiviral activity^37,39^. We found that lipophilic statins were more effective than hydrophilic statins against ZIKV in Vero cells. However, studies using cell lines that allow active transport of statins will have to be performed to be able to determine whether or not hydrophilic statins like PRA and ROS have anti-ZIKV activity. While all the lipophilic statins tested here had anti-ZIKV activity in Vero cells, FLU, LOV, and ATO had generally more superior activity than SIM, MEV, and CER. Although FLU and LOV had generally similar effects on reducing ZIKV titers to nearly 2%, the effective concentration of FLU was lower than that of LOV, suggesting that FLU is more pharmacologically active than LOV. Additionally, while CER showed some anti-ZIKV capacity, its activity was consistently lower than the rest, and seemed to be unaffected by time of addition.

The differences in statin chemical structures probably cause variations in pharmacological and biochemical properties that lead to variations in antiviral capacity. Some studies have revealed that individual statins have distinct interactions with cell membrane models and have different locations within the bilayer at equilibrium. Specifically, CER was found to be embedded within the lipid core of a phospholipid bilayer system, while ATO and SIM localized within the upper hydrocarbon core close to the glycerol backbone of a phospholipid bilayer model^40^. On the other hand, the cyclic portion of FLU has been found to be partially exposed on the polar surface of the phospholipid bilayer^41^. These differences in cell membrane localization may affect the accessibility of these statins to its target. The HMGCR is integrated in the ER membrane, with its active site on the cytosolic side of the membrane. A statin that is closer to the cytosolic side of the ER, such as FLU, may be more available for interaction with HMGCR than a statin that is embedded deep within the lipid core of the ER membrane (e.g. CER). This may therefore partially explain the variations in the statins’ anti-ZIKV activity. These characteristics may be important points for consideration in developing a statin or a new derivative to combat ZIKV. Moreover, our results highlight that testing one representative statin is not sufficient for characterizing the antiviral activities of the class due to differences in the statins’ pharmacological profiles. Additionally, it may be important to note that the effective concentrations in this study were higher than the reported peak plasma concentration (C_max_) in human serum of these statins (C_max_: 0.040 µM for ATO, 0.302 µM for FLU, 0.1–1 µM for MEV, 0.050 µM for LOV and 0.019–0.031 µM for SIM)^42,43^. However, the concentrations used here did not have toxic effects on the cells. Furthermore, the *in vitro* model used here, Vero cells, is of monkey origin and may not fully reflect the conditions for human cells. As such, more studies will have to be performed to test the effects of statins on human *in vitro* models and on animal models for ZIKV.

The complications arising from ZIKV infections (i.e. fetal congenital ZIKV syndrome and GBS in adults) highlight characteristics of anti-ZIKV agents that will maximally benefit those who are most vulnerable. First, a good anti-ZIKV drug must be safe for pregnant women and their fetuses. A study has shown that the use of statins in early pregnancy is not associated with teratogenesis^44^. Although larger studies will have to be conducted, especially if statins are to be considered for use against ZIKV, the results of this study suggest that statins are safe for use by expectant mothers. The capacity to cross the blood-brain barrier and to protect neurons would also be advantageous properties for a prospective anti-ZIKV drug. Statins are, in fact, being considered for treatment of neurodegenerative disorders such as Parkinson’s Disease and Alzheimer’s Disease, showing that they have neuroprotective capacity^45^. Statins, therefore, have advantageous characteristics that may help mitigate the morbidity associated with ZIKV infection.

Despite being currently under control, ZIKV still poses a threat to the world, especially with the increasing risk of its spread to immunologically naïve populations. Therapeutic agents are therefore necessary to prevent infection and mitigate the complications associated with ZIKV. Here, we report that lipophilic statins are able to reduce ZIKV production in Vero cells. Individual statins have varying anti-ZIKV capacities possibly due to differences in physicochemical properties. This study can serve as a starting point for further investigations of statins as a class of drugs in the role of potential anti-ZIKV candidates. The apparent anti-ZIKV activity of statins studied here probably extends to other lipophilic statins and their derivatives, providing us with a wide selection of prospective drugs with varying safety and pharmacological profiles to choose from. Moreover, the ability of statins to decrease ZIKV production *in vitro* indirectly points to the involvement of cholesterol or other products of the MVD pathway in ZIKV biogenesis. This information is an interesting point of study to further our understanding of ZIKV infection mechanisms.

## Methods

### Cells and viruses

African green monkey (Vero; ATCC^®^ CCL-81^TM^) cells were grown in growth medium consisting of Minimal Eagle’s Medium (MEM, Gibco) supplemented with 10% fetal bovine serum, antimycotic antibiotics (Gibco), and L-glutamine (Gibco). ZIKV (ATCC^®^ VR-1838^TM^) was propagated in Vero cells using MEM supplemented with 0.3% bovine serum albumin, antimycotic antibiotics, and L-glutamine (infection medium). The ZIKV particles were harvested at 5 days post-infection (dpi), and culture supernatants were stored at −80 °C until use.

### Reagents

Atorvastatin calcium salt trihydrate (ATO), cerivastatin sodium salt hydrate (CER), fluvastatin sodium hydrate (FLU), pravastatin sodium salt hydrate (PRA), rosuvastatin calcium (ROS), simvastatin (SIM), and chloroquine (CHL) were purchased from Sigma-Aldrich. Lovastatin sodium salt (LOV) and mevastatin sodium salt (MEV) were purchased from Calbiochem. The statins ATO, FLU, PRA, ROS, MEV, LOV, and SIM were reconstituted to 20 mM in dimethyl sulfoxide (DMSO); PRA was reconstituted to 50 mM in DMSO; CER was reconstituted to 20 mM in distilled deionized water. All statins were stored at −20 °C and diluted to working concentrations in infection medium on the same day of use. CHL was dissolved to 100 mM in distilled deionized water, stored at 4 °C, and diluted to the required concentration on the same day of use.

Mouse anti-flavivirus group antigen monoclonal antibody clone D1-4G2-4-15 (4G2 mAb) was purchased from Merck-Millipore (MAB10216) and stored at −20 °C in aliquots prior to use. The secondary antibody, fluorescein isothiocyanate (FITC)-conjugated goat anti-mouse IgG, was purchased from Sigma-Aldrich. The cytotoxicity assay kit, EZ-CYTOX, was purchased from DoGenBio, Co., Ltd. in South Korea.

### Screening for anti-ZIKV activity in dose-response curves

Overnight 96-well plate cultures of Vero cells (1.0 × 10^4^ cells/well) in growth medium were prepared prior to 12-dose response curve assays consisting of half-dilutions starting from 50 µM as the highest dose (n = 3 per dose) of the statins (ATO, CER, FLU, LOV, MEV, PRA, ROS, and SIM). Before any treatment, cells were washed once with phosphate-buffered saline solution (PBS). For the cytotoxicity curve, statins were diluted to required concentrations in infection medium (100 µL/well) and added to Vero cells. All Vero cell controls had 0.05% DMSO except for CER setup, which had no additive. Cells were incubated at 37 °C, at 5% CO_2_ for 5 days. After incubation, EZ-CYTOX (10 µL/well) was added. Cells were incubated at 37 °C, at 5% CO_2_, and the absorbance at 450 nm was read after 3–4 hours. Percent viability was calculated as absorbance of the treated (Abs_treated_) divided by the absorbance of the Vero cell control (Abs_vero_) and then multiplied by 100. Percent cytotoxicity was calculated as 100% minus the % viability. The CC_50_ based on % cytotoxicity was calculated using the 4-parameter logistic curve fitting method of SigmaPlot (Systat Software, San Jose, CA), *p* < 0.0001.

For the activity curve, statins were diluted in infection medium in serial half-dilutions starting from 50 µM as the highest dose and co-introduced with ZIKV (MOI of 0.01) in the Vero cells. Cells were incubated at 37 °C, at 5% CO_2_ for 5 days. After incubation, EZ-CYTOX (10 µL/well) was added. Cells were incubated at 37 °C, at 5% CO_2_, and the absorbance at 450 nm was read after 3–4 hours. Percent protectivity was calculated as the absorbance of the treated (Abs_treated_) minus the absorbance of the infection control (Abs_infected_) over the absorbance of the uninfected control (Abs_vero_) minus Abs_infected_ multiplied by 100% [% Protectivity = (Abs_treated_ − Abs_infected_)/(Abs_vero_ − Abs_infected_) × 100%]. The EC_50_ was calculated using the 4-parameter logistic curve fitting method of SigmaPlot (Systat Software, San Jose, CA), *p* < 0.0001.

### Statin-treatment of ZIKV-infected Vero cells

Vero cells (1.5 × 10^5^ cells/well) were prepared in 12-well plates and incubated overnight at 37 °C in 5% CO_2_. Cells were washed once with PBS and infected with ZIKV (MOI of 0.01) in infection medium. For the treatment groups, ZIKV and lipophilic statins were co-introduced into the Vero cultures. Minimally cytotoxic but effective (around 2 EC_50_ for all but CER) concentrations of lipophilic statins (ATO: 14.69 µM; CER: 91 µM; FLU: 2.97 µM; LOV: 29.18 µM; MEV: 6.78 µM; SIM: 5.09 µM) were used to treat the cells for 96 hours. The positive control was prepared by similarly treating Vero cells with chloroquine (25 µM) in infection medium. Supernatants were harvested at the end of the experiment or at different timepoints, depending on the subsequent quantification assay. Supernatants were stored at −80 °C prior to the assays.

### Timepoint ZIKV titer determination using TCID_50_

Supernatants from the 12-well plate infected and treated Vero cultures were harvested at 6, 24, 48, 72, and 96 hpi (n = 3 per timepoint). The supernatant from each timepoint was serially diluted 10-fold (10^−1^ to 10^−8^) and added to overnight Vero cell cultures (1.0 × 10^4^ cells/well, n = 4 per dilution). Cells were incubated at 37 °C with 5% CO_2_ and observed for cytopathic effects (CPE) 96 hpi compared with uninfected Vero cell controls. The TCID_50_ per timepoint was determined using the Reed-Muench method.

### Determination of ZIKV titer by the plaque assay

Vero cells (~2.5 × 10^5^ cells/well) were prepared in 6-well plates and were incubated at 37 °C with 5% CO_2_ for two days (~90% confluence). The cells were washed with PBS, and then inoculated with 10-fold serially diluted statin-treated ZIKV infection supernatant (n = 3) in infection medium for 1.5 hours at 37 °C, 5% CO_2_. The infection supernatant was removed, and the cells were overlaid with MEM infection medium with bacto-agar (0.9%). Cultures were incubated at 37 °C, 5% CO_2_ for 96 hours, after which the agar overlay was removed. Plaques were stained and fixed in 0.1% crystal violet and 10% formaldehyde. The mean PFU/mL for each statin treatment was calculated, and % infectivity of ZIKV produced from each treatment was calculated as the average titer (PFU/mL) of the treatment divided by the average titer of the ZIKV-infected, untreated control multiplied by 100.

### Immunofluorescence assay

Vero cells (2 × 10^4^ cells/well) in growth medium were incubated overnight in 96-well plates at 37 °C with 5% CO_2_, and cells were washed with PBS. Statins were co-introduced with ZIKV (MOI of 0.1), and cells were incubated at 37 °C, 5% CO_2_ for 48 hours. The cells were washed three times with PBS (all the subsequent wash steps were performed similarly), and fixed and permeabilized for 20 minutes at 4 °C with BD Cytofix/Cytoperm (BD Biosciences). Cells were washed and were blocked using 2% bovine serum albumin overnight at 4 °C. Cells were again washed three times with PBS prior to incubation with 4G2 mAb (1:20 in PBS) for 2 hours at 37 °C. This was followed by a wash step, and then the cells were incubated with FITC-conjugated goat anti-mouse IgG secondary antibody (1:100 in PBS) for 1 hour at 37 °C. Cells were washed and counterstained with DAPI (1:10,000) for 1 minute, then washed again and overlaid with 80% glycerol. Cells were viewed under Carl Zeiss Axio Scope.A1 at 20× magnification, and the images were captured using the AxioVision 4.8 software. Composite images were merged using ImageJ software.

### Time-of-drug-addition assay

Time-of-drug-addition assay was performed based on the study by Delvecchio *et al*.^22^ with some modifications. Overnight Vero cells were prepared in 24-well plates (1.5 × 10^5^ cells/well). The cells were washed once with PBS, and infected with ZIKV (MOI of 5.0) for 1 hour at 4 °C. The cells were washed twice with cold-PBS to remove unbound virus. Infection medium (500 µL/well) was added to the cells, and the drugs diluted in infection medium (500 µL/well) were added to their corresponding working concentrations at different timepoints post-infection (0, 0.5, 3, 12, and 24 hpi; n = 3 per timepoint for each drug). Supernatant was collected at 36 hpi and used for subsequent plaque assay to determine % production of ZIKV.

### Viral internalization assay

The virus internalization assay was derived from the study by Li *et al*.^23^ Vero cell cultures were seeded onto 6-well plates to 95–100% confluence. The cells were washed once with PBS and infected with 300 PFU ZIKV at 4 °C for 1 hour. The cells were washed twice with PBS and the drugs (ATO, FLU, and CHL) diluted to working concentration in infection medium were added (1 mL/well) to the cells (n = 3 per drug). The cells were incubated at 37 °C for 1 hour. The cells were washed twice with PBS and treated with 300 µL citric acid buffer (pH 3.0) for 1 minute. Citric acid buffer was removed, and the cells were immediately overlaid with MEM infection medium with bacto-agar (0.9%) for the subsequent plaque assay.

### Statistical analysis

Student’s *t*-test was used to determine significant differences between pairs of groups (*p < *0.05). One-way analysis of variance (ANOVA) was used to test for significant differences among groups, followed by the Holm-Sidak method to test for differences between pairs of groups using SigmaPlot version 14 (Systat Software, San Jose, CA) (*p* < 0.05).

## Supplementary information

Supplementary information

## Data Availability

Data is presented within the manuscript and supplementary materials.
