# Supplementary material for: Lipophilic statins inhibit Zika virus production in Vero cells
Source: Sci Rep. 2019 Aug 7;9:11461. doi: 10.1038/s41598-019-47956-1 (PMC6685969; doi:10.1038/s41598-019-47956-1)
Supplement: Supplementary file 1 — Supplementary information [file 41598_2019_47956_MOESM1_ESM.pdf]

Supplementary Information

Lipophilic statins inhibit Zika virus production in Vero cells

Erica Españo[1], Jeong-Hyun Nam[1], Eun-Jung Song[1], Daesub Song[1], Chong-Kil Lee[2], Jeong-Ki Kim[1]

[1] Department of Pharmacy, College of Pharmacy, Korea University, Sejong 30019, Republic of Korea

[2] Department of Pharmacy, College of Pharmacy, Chungbuk National University, 194-21 Osongsaengmyeong 1-ro, Osong-eup, Heungdeok-gu, Cheongju, Chungbuk 28160, Republic of Korea

Correspondence and requests for materials should be addressed to J.-K.K. (email: jkfrancis@korea.ac.kr) and C.-K.L. (email: cklee@chungbuk.ac.kr)

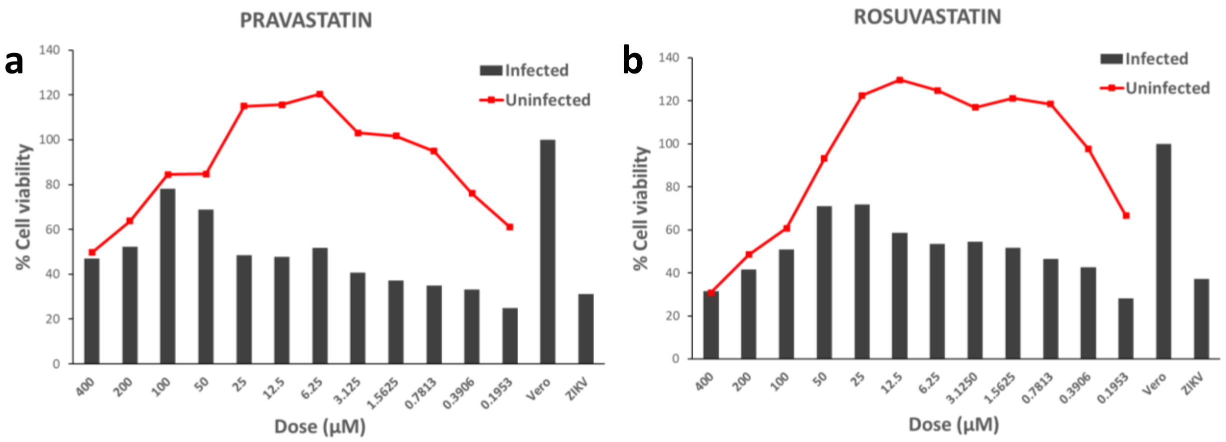

Supplementary Figure S1. Dose-dependent evaluation of the effects of (a) pravastatin and (b) rosuvastatin on the viability of ZIKV-infected and not infected Vero cells. Percent cell viability was calculated as % mean absorbance (450 nm) of the treated samples minus % mean absorbance (450 nm) of the untreated Vero cell control divided by the mean absorbance (450 nm) of the Vero cell control. In the case of pravastatin, minimal effect on the viability of the infected cells was observed till up to 25 µM. The effect increased at around 50 µM; however, this was equivalent to the viability observed in the non-infected Vero cells. Percent cell viability values of Vero cells from 50 to 400 µM pravastatin were similar in both the infected and non-infected cells that the apparent activity of pravastatin may be attributed to its effect on Vero cells rather than to its effect on ZIKV infection. A similar trend could be observed for the effect of rosuvastatin starting at 25 to 400 µM on infected and not infected Vero cells. Graphs also show the % cell viability of controls: uninfected, untreated cells (Vero), and untreated, infected cells (ZIKV).

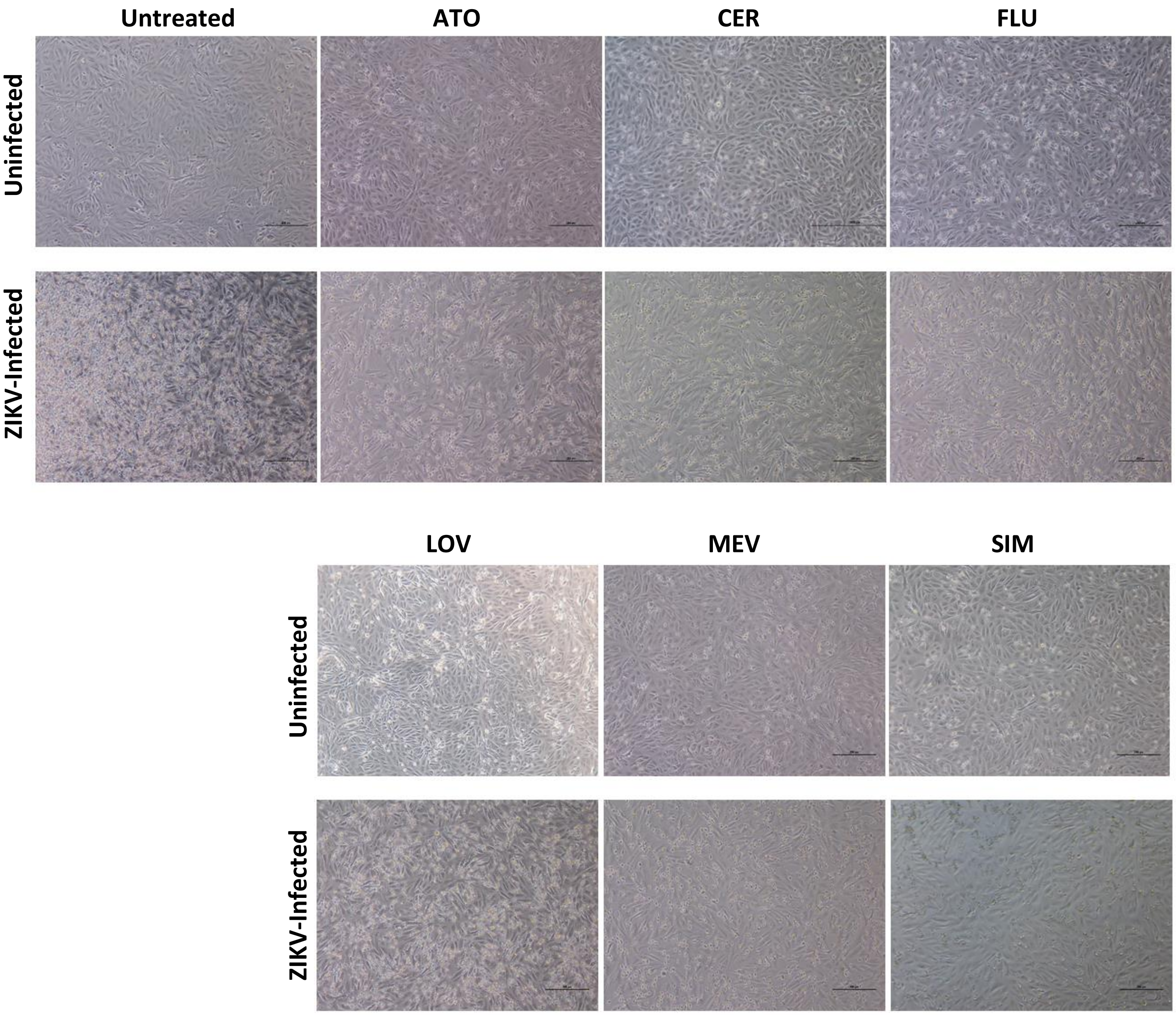

Supplementary Figure S2. Phenotype of Vero cell monolayer cultures uninfected vs. infected with ZIKV, treated with lipophilic statins. Morphology of Vero cell cultures 96 hours post-treatment with lipophilic statins at the evaluated concentrations were observed for cytopathic effects (CPE) and relative health. Morphology of uninfected, treated cell cultures were similar to that of the uninfected, untreated control indicating that the lipophilic statins at the evaluated doses had little to no effect on growth of the cells. Cytopathic effects could be observed in the untreated ZIKV-infected control as cell rounding and detachment. Similar CPE was observed in some of the treated ZIKV-infected controls, particularly in the LOV setup, but were not as prominent as in the ZIKV-infected control.
